# Synthetic RIG-I agonist-mediated cancer immunotherapy synergizes with MAP kinase inhibition against BRAF-mutated melanoma

**DOI:** 10.1016/j.omtn.2024.102283

**Published:** 2024-07-19

**Authors:** Christian Grützner, Yu Pan Tan, Patrick Müller, Thais M. Schlee-Guimaraes, Marius Jentzsch, Jonathan L. Schmid-Burgk, Marcel Renn, Rayk Behrendt, Gunther Hartmann

**Affiliations:** 1 Institute for Clinical Chemistry and Clinical Pharmacology, University Hospital Bonn, Bonn, Germany; 2 Department of Oncology, Hematology, Immuno-Oncology and Rheumatology, University Hospital Bonn, Bonn, Germany; 3 Center for Integrated Oncology Aachen Bonn Cologne Düsseldorf (CIO ABCD), Bonn, Germany

**Keywords:** MT: Oligonucleotides: Therapies and Applications, RIG-I, interferon, MAP kinase inhibitor, melanoma, BRAF, intratumoral therapy

## Abstract

The implementation of targeted molecular therapies and immunotherapy in melanoma vastly improved the therapeutic outcome in patients with limited efficacy of surgical intervention. Nevertheless, a large fraction of patients with melanoma still remain refractory or acquire resistance to these new forms of treatment, illustrating a need for improvement. Here, we report that the clinically relevant combination of mitogen-activated protein (MAP) kinase pathway inhibitors dabrafenib and trametinib synergize with RIG-I agonist-induced immunotherapy to kill BRAF-mutated human and mouse melanoma cells. Kinase inhibition did not compromise the agonist-induced innate immune response of the RIG-I pathway in host immune cells. In a melanoma transplantation mouse model, the triple therapy outperformed individual therapies. Our study suggests that agonist-induced activation of RIG-I with its synthetic ligand 3pRNA could vastly improve tumor control in a substantial fraction of patients with melanoma receiving MAP kinase inhibitors.

## Graphical abstract

**Figure:**
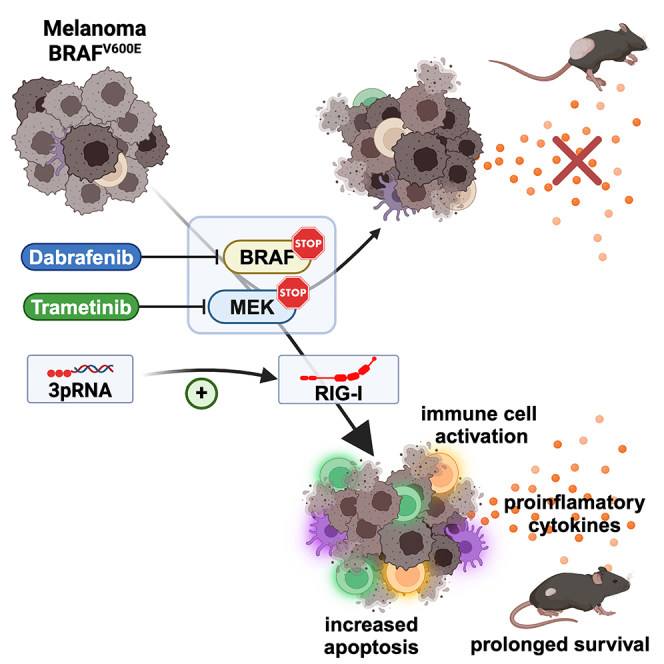

## Teaser

Grützner and colleagues demonstrate that in BRAF-mutated melanoma, RIG-I agonist-induced cancer immunotherapy combined with standard-of-care MAP kinase pathway inhibitors dabrafenib and trametinib outperforms the individual therapeutic efficacy of each inhibitor. Their study showcases how including RIG-I agonists in combination therapies can overcome primary or acquired resistances of melanoma to monotherapies.

## Introduction

Melanoma is a metastasizing skin cancer arising from pigment-producing melanocytes that has seen a concerning rise in incidence over the last decades.^1^^,^^2^^,^^3^ About half of all melanomas present with a gain-of-function mutation in the mitogen-activated protein kinase (MAPK) / extracellular signal-regulated kinase (ERK) pathway, specifically in the kinase BRAF, promoting proliferation and survival.^4^^,^^5^ More than 90% of all clinically recorded BRAF-mutated melanomas carry a V600E mutation,^6^^,^^7^ which can be targeted pharmacologically by inhibition of BRAF itself or the downstream kinase MEK with drugs like dabrafenib and trametinib, respectively. This combination represents the current standard of care for BRAF-mutated melanoma in adjuvant or definitive treatment settings.^8^^,^^9^^,^^10^^,^^11^ BRAF/MEK inhibition has pleiotropic immunomodulatory effects like increased CD8^+^ T cell counts, increased major histocompatibility complex class I, and increased tumor antigen expression in melanoma and a concomitant reduction of the immunosuppressive tumor micromilieu.^12^^,^^13^^,^^14^ Despite of high initial response rates, treatment success is compromised by early resistance to targeted therapy, with a 5-year overall survival of about 30%.^15^ To improve these drawbacks, first trials reported promising results from combination therapies of BRAF/MEK inhibitors and immune checkpoint inhibitors like pembrolizumab, nivolumab, and ipilimumab,^16^^,^^17^ but they also observed severe toxicities, especially for ipilimumab-based combinations.^18^^,^^19^ This highlights that immune modulatory therapy can restore the response to compromised BRAF/MEK-inhibitor-based treatment regime.

Intratumoral activation of the cytoplasmic double-stranded RNA (dsRNA) sensor retinoic acid-inducible gene I (RIG-I) alone elicits a potent anti-tumor immune response resulting in melanoma control through the concurrent induction of a pro-inflammatory immune response and programmed cell death of tumor cells.^20^^,^^21^^,^^22^ We recently reported that radioresistant p53-positive tumors can be controlled by intratumoral activation of RIG-I.^23^ Here, we show that systemic BRAF/MEK inhibition synergizes with intratumoral RIG-I activation, inducing an immunogenic cell death in BRAF-mutated human and murine melanoma cells. *In vivo*, BRAF/MEK inhibitors act in concert with RIG-I ligands to synergistically improve the survival of melanoma-bearing mice. Our data suggest that intratumoral activation of RIG-I has the potential to improve cancer immunotherapy in almost half of patients with melanoma susceptible to BRAF/MEK inhibition.

## Results

### Activation of RIG-I induces a pro-inflammatory signature and increased cell death of BRAF/MEK-inhibited melanoma

**Figure 1:**
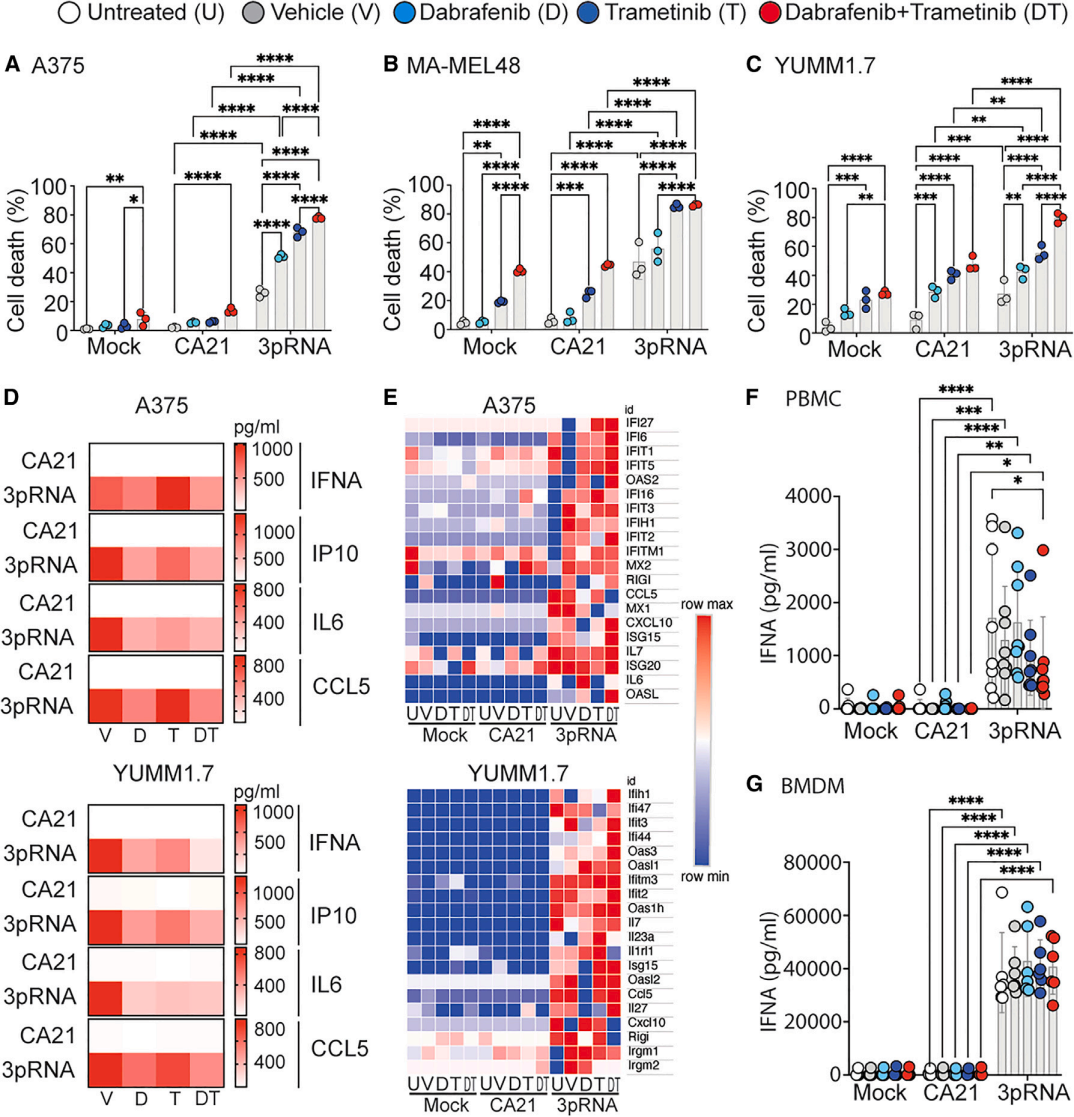
Combined BRAF/MEK inhibition synergizes with intratumoral RIG-I activation to kill melanoma cells and activate immune cells In all images, treatment was as follows: untreated (U), DMSO (= vehicle [V]), 1,000 nM dabrafenib (D), 100 nM trametinib (T), and 1,000 nM dabrafenib +100 nM trametinib (DT). For RIG-I stimulation: untreated (MOCK), 1,000 ng/mL CA21 control RNA, 1,000 ng/mL 3pRNA. (A–C) Percentage of dead cells was quantified by flow cytometry after 48 h. Each data point represents one independent experiment shown as the mean of two technical replicates per experiment. Mean of the independent experiments ± SD is shown. (D) The indicated cytokines in the supernatant of A375 (top) and YUMM1.7 (bottom) cells after 24 h. Data represent the mean of five independent experiments with two technical replicates per experiment. (E) Transcription of interferon-regulated genes in A375 (top) and YUMM1.7 (bottom) cells was measured after 24 h by full-length mRNA sequencing. (F–G) IFNA protein in the supernatant of PBMCs (top) and bone marrow-derived macrophages (BMDMs) (bottom) was quantified by ELISA. Only significant results indicated. Each data point represents one biological donor. Per independent experiment, two technical replicates of two donors were analyzed. Mean of all donors ± SD is shown. Statistics for (A)–(C), (F), and (G) were calculated using two-way ANOVA with Tukey post hoc test. Only significant differences are labeled. ∗*p* < 0.05, ∗∗*p* < 0.01, ∗∗∗*p* < 0.001, and ∗∗∗∗*p* < 0.0001.

To investigate if the clinically relevant melanoma therapy of combined BRAF and MEK inhibition sensitizes cancer cells for the treatment with the RIG-I ligand 3pRNA, we treated human A375 (BRAF V600E mutated), Ma-Mel-48 (H-Ras G13I), and murine YUMM1.7 (BRAF V637E is a homolog to human V600E, V637E is sensitive to dabrafenib^24^) melanoma cell lines with dabrafenib and trametinib, alone or in combination. Dabrafenib alone did not increase the frequency of Annexin V- or 7AAD-positive A375 and Ma-Mel-48 cells, while trametinib alone had a moderate effect on the viability of Ma-Mel-48 cells (Figures 1A and 1B). Combined BRAF and MEK inhibition or transfection of 3pRNA alone only slightly increased the frequency of dead A375 cells but killed almost half of Ma-Mel-48 melanoma cells (Figures 1A and 1B). The effect was synergistic when stimulation of RIG-I was paired with dabrafenib or trametinib alone, and it was strongest when all treatments were combined (Figures 1A and 1B). The findings were replicated in murine YUMM1.7 melanoma cells (Figure 1C).

Next, we screened the supernatant of BRAF/MEK-inhibited and control melanoma cells 24 h after 3pRNA stimulation for cytokine expression. We observed high levels of interferon alpha (IFNA), 10kDa interferon gamma-induced protein (IP10), interleukin-6 (IL6), and cc-chemokine ligand 5 (CCL5) proteins in the supernatant (Figure 1D) as well as a pro-inflammatory transcriptional signature (Figure 1E) in A375 and YUMM1.7 melanoma cells after transfection of 3pRNA, which was largely unaffected by dabrafenib or trametinib (Figures 1D and 1E). To investigate the effect of dabrafenib or trametinib on the activity of the RIG-I pathway in immune cells, we isolated human peripheral blood mononuclear cells (PBMCs) and murine bone marrow-derived macrophages and stimulated them with 3pRNA in the presence or absence of dabrafenib, trametinib, or their combination. Aside from a slightly diminished IFNA secretion in PBMCs in response to 3pRNA transfection, BRAF/MEK inhibition did not compromise the activity of RIG-I in professional primary immune cells (Figures 1F and 1G).

### Combined BRAF/MEK inhibition overcomes resistance to 3pRNA treatment *in vivo*

To test the effect of RIG-I stimulation in combination with BRAF/MEK inhibition *in vivo*, we used a spontaneous melanoma model, in which tamoxifen-induced Cre-mediated expression of the BRAF V600E variant and concomitant deletion of the Pten tumor suppressor induces melanoma within 2–12 months after treatment.^25^ We observed visible tumors in a substantial fraction of tamoxifen naive mice, which is in line with latest reports provided by the vendor (see Jax Strain 013590) but complicated harmonized treatment initiation of mice. Despite the leakiness, we induced tumor development in mice that had no visible tumor at the age of 6–8 weeks and started therapy 3 weeks later. Tumor progression was delayed by intratumoral 3pRNA injection in mice treated with dabrafenib and trametinib in combination, but the difference failed to reach statistical significance (Figure S1, *p* = 0.0722, unadjusted Gehan-Breslow-Wilcoxon test).

**Figure 2:**
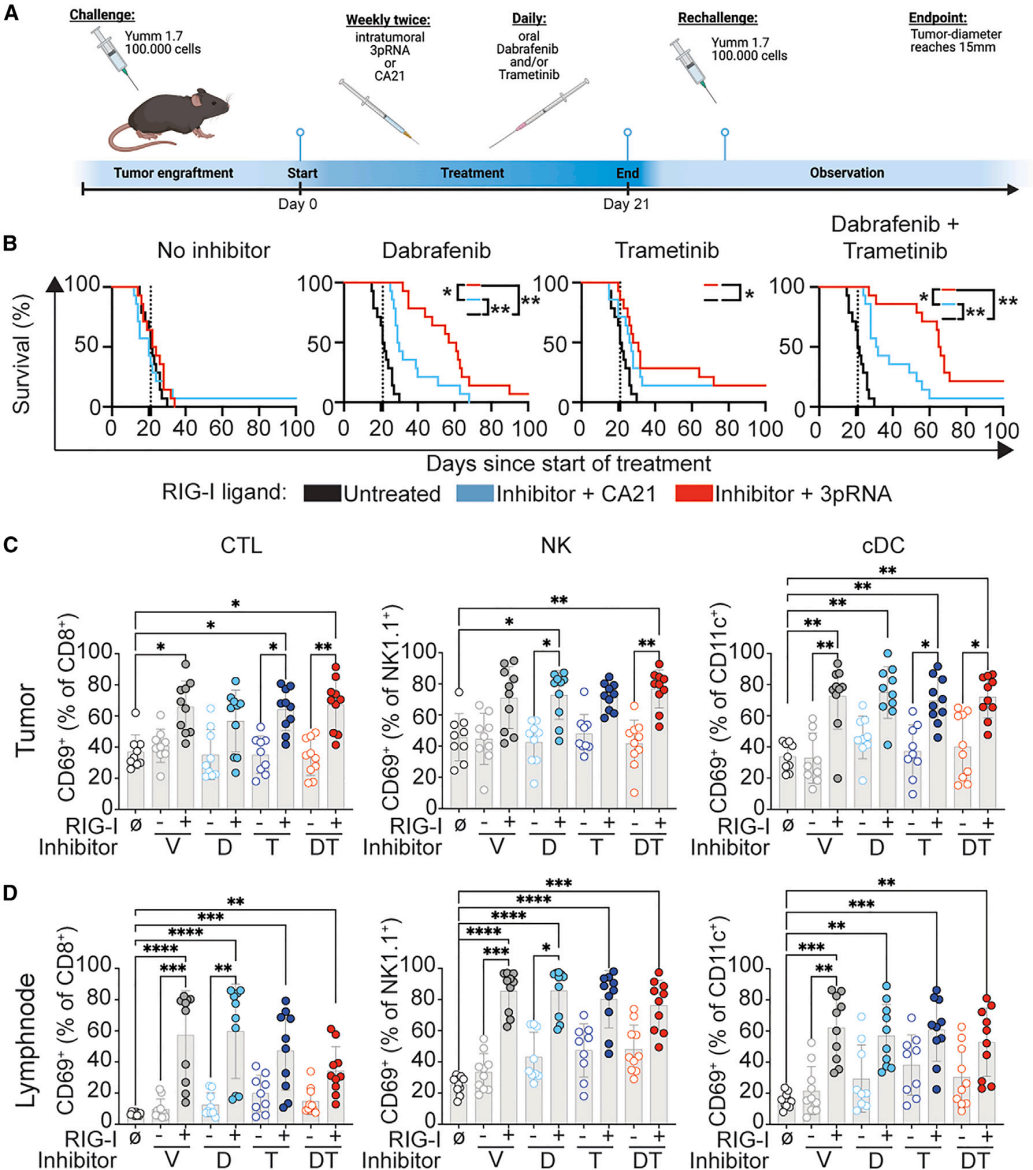
Synergistic anti-tumor immunity against BRAF-mutated melanoma *in vivo* through combined RIG-I stimulation and BRAF/MEK inhibition (A) Timeline of YUMM1.7 transplantation, treatment, and analysis. (B) Overall survival of tumor-bearing mice treated as indicated. Three independent experiments were performed with a total *n* = 14 in each group. Log-rank Mantel-Cox test with Holm-Šídák correction for 12 multiple comparisons. ∗*p* < 0.05 and ∗∗*p* < 0.01. (C and D) Frequency of activated (CD69^+^) among CD45^+^CD4^−^CD8^+^ CTLs, CD45^+^NK1.1^+^ NK cells, and CD45^+^CD11b^+^CD11c^+^MHC class II^+^ cDCs in the tumor (C) and the tumor-draining lymph node (D) 16 h after a single treatment. Mean ± SD is shown. Kruskal-Wallis test with Dunn’s correction for 16 multiple comparisons. ∗*p* < 0.05, ∗∗*p* < 0.01, ∗∗∗*p* < 0.001, and ∗∗∗∗*p* < 0.0001. Only significant results indicated.

To overcome the uncontrollable biological variability introduced by Cre leakiness in the spontaneous tumor model, we turned toward a transplantation model using YUMM1.7 melanoma cells (Figure 2A). RIG-I agonist treatment started at a tumor diameter of maximal 3 mm twice a week for 21 days and daily treatment with dabrafenib and trametinib. At days 11–15 after treatment discontinuation, all surviving mice were re-challenged with YUMM1.7 cells injected into the contra-lateral side (Figure 2A). When analyzing the overall survival, irrespective of the re-challenge, YUMM1.7 tumors were resistant to trametinib and 3pRNA monotherapies but, as expected, sensitive to dabrafenib (Figure 2B). 3pRNA stimulation improved the survival of mice that received dabrafenib, but not trametinib monotherapy when compared to injections of CA21 control RNA. Strikingly, the clinically applied combination of dabrafenib and trametinib synergized most potently with additional stimulation of RIG-I, resulting in the strongest improvement in survival (Figure 2B). Censoring all events that are associated with the tumor re-challenge of mice that survived the therapy of the primary tumor confirmed the results of the overall analysis (Figure S1C). In contrast, analyzing only events that can be attributed to the contra-lateral tumor injection revealed no effect on survival in any treatment (Figure S1C). However, the latter analysis suffers from decreased power due to the low number of mice that survived the primary tumor.

Next, we analyzed the effect of the different therapeutic combinations on tumor-infiltrating immune cells and in tumor-draining lymph nodes 16 h after a single intratumoral injection of 3pRNA. Stimulation with the RIG-I agonist increased the frequencies of activated (CD69^+^) CD8^+^ cytotoxic T cells (CTL), natural killer (NK), and conventional dendritic cells (cDCs) in YUMM1.7 tumors (Figure 2C). Additional treatment with dabrafenib, trametinib, or both inhibitors combined did not suppress 3pRNA-induced local inflammation (Figure 2C). Similarly, higher frequencies of activated CD8^+^ cells, NK cells, and cDCs were found in the tumor-draining lymph nodes, irrespective of the treatment with dabrafenib and trametinib (Figure 2D).

Collectively, we show that intratumoral injection of a synthetic RIG-I ligand led to the recruitment of activated immune cells into the tumor microenvironment and tumor-draining lymph nodes, which was unaltered by treatment with dabrafenib and trametinib. Most importantly, in this setting, intratumoral activation of RIG-I synergistically improved the survival of mice carrying BRAF-mutated melanoma.

## Discussion

Here, we show that intratumoral activation of intracellular nucleic acid sensor RIG-I^20^^,^^21^ enhances the immune response and cell death of BRAF- and MEK-mutated melanoma cells. The effect was even stronger when combined with melanoma standard-of-care BRAF/MEK inhibitors dabrafenib and trametinib, respectively. The study supports previous observations, which suggested that inhibition of MAPKs sensitizes BRAF-mutated melanoma cells to RIG stimulation through the induction an IRF1-dependent pro-inflammatory program.^26^ However, in clinical care of melanoma, BRAF and MEK inhibitors are strictly used in combination, while dabrafenib monotherapy is a rare exception. We now also show that the clinically applied combination of BRAF/MEK inhibition does not compromise the therapeutic effects of intratumoral RIG-I activation in *in vivo* melanoma models: first, 3pRNA directly acts within the tumor cells, likely inducing a caspase-dependent cell death as observed previously,^23^ thereby reducing tumor burden. Second, activation of RIG-I induced a pro-inflammatory response in the tumor cells, thereby attracting immune cells to the site of the tumor. Third, RIG-I agonists directly activate professional antigen-presenting cells of the host that reside in the tumor, which subsequently instruct adaptive anti-tumor immunity. We observed that both the tumor cell-intrinsic and immune cell-intrinsic RIG-I responses are functional under combined BRAF/MEK inhibitor therapy.

*In vivo*, intratumoral RIG-I stimulation could overcome the resistance of YUMM1.7 to trametinib monotherapy, and it significantly prolonged survival under dabrafenib therapy alone and when combined with trametinib. Of note, YUMM1.7 melanoma are by far less immunogenic when compared to RIG-I-sensitive YUMMER 1.7 melanoma,^27^ and we provide evidence that combined BRAF/MEK inhibition renders even YUMM1.7 melanoma susceptible to RIG-I agonist-based immunotherapy. We previously observed that RIG-I stimulation overcomes radiotherapy resistance in p53-positive melanoma, suggesting that RIG-I-driven innate immune activation in the tumor microenvironment might represent a general strategy to enhance various melanoma therapies.^23^

Taken together, we show that intratumoral stimulation of RIG-I with its synthetic ligand 3pRNA in combination with the clinically relevant BRAF/MEK double inhibition outperforms the therapeutic effect of the individual therapies against BRAF-mutated melanoma *in vivo*. Our data suggest that this combination, which potentially improves the outcome for almost half of patients with melanoma, could be a major asset to the toolbox of melanoma therapy.

## Material and methods

Standard procedures are described in the supplemental information.

### Mouse experiments

All mouse experiments were approved by the Landesamt für Natur, Umwelt und Verbraucherschutz NRW (81-02.04.2020.A156). B6.Cg-Tg(Tyr-cre/ERT2)13Bos *Braf*^*tm1Mmcm*^ *Pten*^*tm1Hwu*^/BosJ (BRaf^CA^, Pten^loxP^, Tyr:CreER^T2^) were purchased from Jackson Laboratory (JAX 013590).^25^ Mice of both sexes were topically treated with 2 μL of 5 mM 4-hydroxytamoxifen (Sigma-Aldrich) at the age of 6–8 weeks and observed as reported in the main text. For transplantation of YUMM1.7 cells, 100,000 cells in 50 μL of Dulbecco’s balanced salt solution (PBS, Thermo Fisher Scientific) were intracutaneously injected in the flank of C56BL6/J mice. When tumor diameters reached 2–3 mm, mice were randomly assigned to treatment groups, and tumor diameter was measured daily using a caliper.

BRAF inhibitor dabrafenib (mesylate) and MEK inhibitor trametinib (both MedChem Express) were suspended in an aqueous solution containing 0.5% (w/v) hydroxypropyl-methylcellulose (Sigma-Aldrich) and 0.2% (v/v) Tween 80 (Sigma-Aldrich). Inhibitors were administered by oral gavage in a final volume of 100 μL. To stimulate RIG-I 3pRNA or CA21, control RNA was complexed with jetPEI (Polyplus Transfection) according to the manufacturer’s protocol and injected intratumorally.

### Treatment of cells

For RIG-I stimulation, a chemically synthesized dsRNA with a 3p end, as previously described, was used.^28^ CA21 control RNA was synthesized at Biomers (5′-CACACACACACACACACACAC-3′). For *in vitro* transfection, RNA was complexed to Lipofectamine 2000 (Invitrogen) according to the manufacturer’s protocol (Thermo Fisher Scientific).^23^

For *in vitro* experiments, dabrafenib (MedChem Express) and trametinib (Selleck Chemicals) were dissolved in dimethyl sulfoxide (DMSO) (Carl Roth) and added at indicated concentrations. DMSO at the highest used concentration served as the vehicle control.

### Statistical analysis

Statistical analysis was calculated with Prism 9 (v.9.4.1) software (GraphPad Software). The respective tests are indicated in the figure legends, with *p* < 0.05 considered to be statistically significant.

## Data and code availability

All primary data are available upon request. Gene expression data have been deposited at the GEO database.

## Supplemental information

Document S1. Figure S1 and supplemental materials and methods

Document S2. Article plus supplemental information
