# Supplementary material for: Synthetic RIG-I agonist-mediated cancer immunotherapy synergizes with MAP kinase inhibition against BRAF-mutated melanoma
Source: Mol Ther Nucleic Acids. 2024 Jul 19;35(3):102283. doi: 10.1016/j.omtn.2024.102283 (PMC11334831; doi:10.1016/j.omtn.2024.102283)
Supplement: Document S1. Figure S1 and supplemental materials and methods [file mmc1.pdf]

OMTN, Volume *35*

Supplemental information

Synthetic RIG-I agonist-mediated cancer immunotherapy synergizes with MAP kinase inhibition against BRAF-mutated melanoma

Christian Grützner, Yu Pan Tan, Patrick Müller, Thais M. Schlee-Guimaraes, Marius Jentzsch, Jonathan L. Schmid-Burgk, Marcel Renn, Rayk Behrendt, and Gunther Hartmann

Supplemental Figure

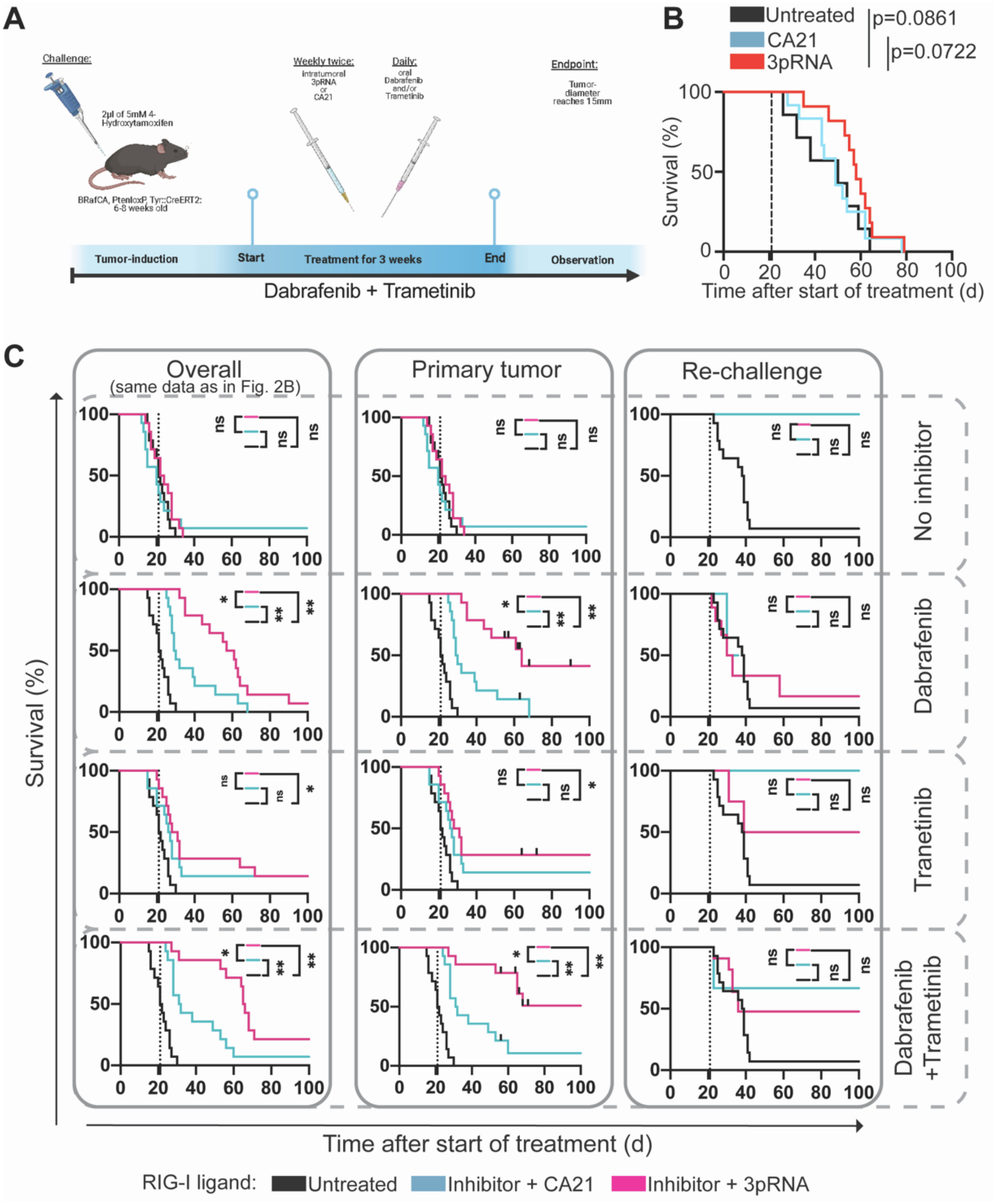

Figure S1: In vivo effect of 3pRNA in combination with Dabrafenib and Trametinib on the survival of melanoma-bearing mice. A. Overview of the experimental design for induction and treatment of Tamoxifen-induced Braf/Pten-mutated melanoma. B. Kaplan-Meyer analysis of Tamoxifen-treated mice that received Dabrafenib and Trametinib and 3pRNA (n=11), CA21-control RNA (n=12) or were left untreated (n=7). Treatment was stopped at day 21 (dotted line). Statistics unadjusted Gehan-Breslow-Wilcoxon test. C) Survival analysis of mice that were inoculated with YUMM1.7 melanoma cells and treated according to the scheme shown in Fig. 2A. Three independent experiments were performed with a total n=14 in each group. Survival curves were generated looking at the overall survival ("Overall", display identical to Fig. 2B), taking only events that could be attributed to the primary tumor into account ("Primary tumor") or only considering events that were presumably caused by the re-challenge tumor ("Re-challenge"). Log-rank Mantel-Cox test with Holm-Šídák correction for 12 multiple comparisons. * - p<0.05; ** - p<0.01.

Supplemental Methods

Cell lines

Human A375 melanoma cell line was kindly provided by Michael Hölzel (University Hospital Bonn, Germany) and Ma-Mel-48 melanoma were kindly provided by the Department of Dermatology at University Hospital Bonn. Murine Yale University Mouse Melanoma - YUMM1.7 was purchased from ATCC [1]. All cell lines were incubated at 37°C 5% CO2 and regularly checked for mycoplasma contamination. A375 cell line was cultured in DMEM with 10 % (v/v) fetal calf serum (FCS) and 100 units penicillin and 100 µg streptomycin per mL Media (p/s) (all Thermo Fisher Scientific). Ma-Mel-48 cell were cultured in RPMI (Thermo Fisher Scientific) containing the same supplements as A375. YUMM cell lines were cultured in DMEM/F-12, GlutaMAX™ (Thermo Fisher Scientific) supplemented with FCS, p/s and non-essential amino acids (Thermo Fisher Scientific).

Peripheral blood mononuclear cell experiments (PBMC):

All experiments on human samples were approved by local ethical committee (Ethikkommission der Medizinischen Fakultät Bonn - File Reference: 515/20). PBMC were isolated by Ficoll (GE Healthcare/Cytiva). Freshly isolated cells were plated in 96 well flat bottom cell culture plates at concentration of 200.000 per well in RPMI 1640 Medium with 10% FCS and 100 units penicillin and 100 µg streptomycin per mL. Cells were stimulated as described in the main text.

Murine bone marrow derived macrophages (BMDM):

Murine bone marrow derived macrophages (BMDM) were differentiated from C56BL/6N whole bone marrow cells for seven days in RPMI + 30% L929 supernatant. 100.000 cells were seeded in 96-well flat bottom tissue culture plates in RPMI as described above RPMI + 15% L929 supernatant and stimulated as described above. All experiments on mouse organs were approved by lokal ethical committee.

Apoptosis assessment by flowcytometry

20.000 (A375/ Ma-Mel-48) or 10.000 (YUMM1.7) cells per well were plated in 96-well flat bottom tissue culture plates. The next day cells were stimulated and/or co-incubated with inhibitors at indicated concentrations. For assessment of apoptosis cells were trypsinized (0.25%, Thermo Fischer Scientific) and stained with Annexin V Alexa Fluor® 647 (BioLegend) at dilution of 1:30 in Annexin binding buffer (10 mM HEPES, 140 mM NaCl, 2.5 mM $CaCl_2$) for 15 min in the dark. Cells were washed in FACS buffer and kept resuspended annexin binding buffer. Shortly before measurement via Attune NxT flow cytometer (Thermo Fisher Scientific) 7-Aminoactinomycin D (Enzo Life Sciences) at final concentration of 1,25 µg/mL was added.

Enzyme linked immuno assay (ELISA):

Commercial ELISAs (Human IP-10, BD; human IFNA, Thermo Fisher) were performed using half of the amounts of chemicals recommended by the manufacturers.

A self-made murine IFNA ELISA was performed in half-area 96 well-microplate (clear, microlon, high binding from Greiner Bio-One) utilizing anti-Mouse IFN-alpha (clone RMMA-1 (MAb)), mouse IFN-alpha A and anti-Mouse IFN-alpha (rabbit Serum (PAb)) (all PBL Assay Science).

Muliplex cytokine Assays:

LEGENDplex™ Assays (BioLegend) were used according to manufacturer's recommendations but run in a 384-well assay plate with volumes adjusted accordingly as previously described [2]. Human cytokines were quantified with the COVID-19 Cytokine Storm Panel (14-plex) (BioLegend 741089) and murine cytokines using Mouse Anti-Virus Response Panel (13-plex) (BioLegend 740622) were used. Data were analyzed using cloud-based LEGENDplex™ Data Analysis Software Suite.

Flow cytometry:

Tumors were desintegrated in PBS containing 1mg/mL Collagenase D (Roche) and 5 % FCS for 20 minutes at 37°C and afterwards passed through a 70 µm cell strainer. Tumor draining lymph nodes were passed through a cell strainer. Zombie Aqua™ Fixable Viability Kit (1:500 in PBS, BioLegend) was added for 20 min at room temperature followed by Fc block using CD16/32 antibodies (1:200, eBioscience) for another 20 mins. Surface marker staining was performed for 20 minutes at room temperature in the dark with following antibodies all diluted at 1:200 in FACS-buffer (PBS with 10% FCS, 2 mM EDTA and 0.05% sodium azide). Results were calculated based on the following gatings using FlowJo software version 10.8.1(BD). 1. pre-gating: SSC:FSC > FSC-H:FSC-A > CD45-PerCP^{+}:Zombie-negative. For identification of immune cell subsets subsequent gatings were applied for activated CD8-T-cells: CD69-AF647^{+} : CD8a-BV785^{+}; activated NK-cells: CD69-AF647^{+} : NK1.1-BV650^{+}., and for cDCs: CD11b-BV650^{+} : FSC > CD11c-BV421^{dim} : MHCII-AF488^{+}. In these subsets also the CD86-BV785 signal was quantified.

RNA-Seq-analysis:

RNA was extracted with RNeasy Mini Kit (Qiagen) used according to the manufacturers protocol. mRNA was purified by poly-A enrichment using NEBNext® Poly(A) mRNA Magnetic Isolation Module. For library preparation NEBNext® Ultra™ II Directional RNA Library Prep with Sample Purification Beads with NEBNext® Multiplex Oligos for Illumina® were used. Sequencing was performed on an Illumina NextSeq 2000 using a P2 100-cycle kit. Sequencing reads were aligned to the human (GRCh38) and Mus musculus (GRCm39) reference genome using STAR (Dobin et al., 2013). and quantified with HTSeq2.0 (G Putri, S Anders, PT Pyl, JE Pimanda, F Zanini Analysing high-throughput sequencing data in Python with HTSeq 2.0 [3]. Expression analysis was performed with the statical R-package edgeR [4] Library sizes across samples were normalized using TMM (trimmed mean of M values).
Data have been deposited in the GEO database under the accession numbers GSE269008 and GSE268982 for mouse and human cell lines, respectively. Heatmaps display log-counts-per-million values of the indicated transcripts after normalization. Heatmaps were generated using the mighty Morpheus (https://software.broadinstitute.org/morpheus).

References for supplemental methods

1. Meeth, K., Wang, J.X., Micevic, G., Damsky, W., and Bosenberg, M.W. (2016). The YUMM lines: a series of congenic mouse melanoma cell lines with defined genetic alterations. Pigment Cell Melanoma Res. *29*, 590–597. https://doi.org/10.1111/pcmr.12498.

2. Lambing, S., Holdenrieder, S., Müller, P., Tan, Y.P., Hagen, C., Garbe, S., Schlee, M., Boorn, J.G. van den, Bartok, E., Hartmann, G., et al. (2022). RIG-I immunotherapy overcomes radioresistance in p53-positive malignant melanoma. Preprint at bioRxiv, https://doi.org/10.1101/2021.10.16.464638 https://doi.org/10.1101/2021.10.16.464638.

3. Putri, G.H., Anders, S., Pyl, P.T., Pimanda, J.E., and Zanini, F. (2022). Analysing high-throughput sequencing data in Python with HTSeq 2.0. Bioinformatics *38*, 2943–2945. https://doi.org/10.1093/bioinformatics/btac166.

4. Robinson, M.D., McCarthy, D.J., and Smyth, G.K. (2010). edgeR: a Bioconductor package for differential expression analysis of digital gene expression data. Bioinforma. Oxf. Engl. *26*, 139–140. https://doi.org/10.1093/bioinformatics/btp616.
