# Supplementary material for: Synthetic RIG-I agonist-mediated cancer immunotherapy synergizes with MAP kinase inhibition against BRAF-mutated melanoma
Source: Mol Ther Nucleic Acids. 2024 Jul 19;35(3):102283. doi: 10.1016/j.omtn.2024.102283 (PMC11334831; doi:10.1016/j.omtn.2024.102283)
Supplement: Document S2. Article plus supplemental information [file mmc2.pdf]

Molecular Therapy
Nucleic Acids
Brief Report

Synthetic RIG-I agonist-mediated cancer immunotherapy synergizes with MAP kinase inhibition against BRAF-mutated melanoma

Christian Grützner,[1,2,3] Yu Pan Tan,[1] Patrick Müller,[1] Thais M. Schlee-Guimaraes,[1] Marius Jentzsch,[1] Jonathan L. Schmid-Burgk,[1] Marcel Renn,[1] Rayk Behrendt,[1,4] and Gunther Hartmann[1,4]

[1]Institute for Clinical Chemistry and Clinical Pharmacology, University Hospital Bonn, Bonn, Germany; [2]Department of Oncology, Hematology, Immuno-Oncology and Rheumatology, University Hospital Bonn, Bonn, Germany; [3]Center for Integrated Oncology Aachen Bonn Cologne Düsseldorf (CIO ABCD), Bonn, Germany

The implementation of targeted molecular therapies and immunotherapy in melanoma vastly improved the therapeutic outcome in patients with limited efficacy of surgical intervention. Nevertheless, a large fraction of patients with melanoma still remain refractory or acquire resistance to these new forms of treatment, illustrating a need for improvement. Here, we report that the clinically relevant combination of mitogen-activated protein (MAP) kinase pathway inhibitors dabrafenib and trametinib synergize with RIG-I agonist-induced immunotherapy to kill BRAF-mutated human and mouse melanoma cells. Kinase inhibition did not compromise the agonist-induced innate immune response of the RIG-I pathway in host immune cells. In a melanoma transplantation mouse model, the triple therapy outperformed individual therapies. Our study suggests that agonist-induced activation of RIG-I with its synthetic ligand 3pRNA could vastly improve tumor control in a substantial fraction of patients with melanoma receiving MAP kinase inhibitors.

INTRODUCTION

Melanoma is a metastasizing skin cancer arising from pigment-producing melanocytes that has seen a concerning rise in incidence over the last decades.[1–3] About half of all melanomas present with a gain-of-function mutation in the mitogen-activated protein kinase (MAPK) / extracellular signal-regulated kinase (ERK) pathway, specifically in the kinase BRAF, promoting proliferation and survival.[4,5] More than 90% of all clinically recorded BRAF-mutated melanomas carry a V600E mutation,[6,7] which can be targeted pharmacologically by inhibition of BRAF itself or the downstream kinase MEK with drugs like dabrafenib and trametinib, respectively. This combination represents the current standard of care for BRAF-mutated melanoma in adjuvant or definitive treatment settings.[8–11] BRAF/MEK inhibition has pleiotropic immunomodulatory effects like increased $CD8^+$ T cell counts, increased major histocompatibility complex class I, and increased tumor antigen expression in melanoma and a concomitant reduction of the immunosuppressive tumor micromilieu.[12–14] Despite of high initial response rates, treatment success is compromised by early resistance to targeted therapy, with a 5-year overall survival of about 30%.[15] To improve these drawbacks, first trials reported promising results from combination therapies of BRAF/MEK inhibitors and immune checkpoint inhibitors like pembrolizumab, nivolumab, and ipilimumab,[16,17] but they also observed severe toxicities, especially for ipilimumab-based combinations.[18,19] This highlights that immune modulatory therapy can restore the response to compromised BRAF/MEK-inhibitor-based treatment regime.

Intratumoral activation of the cytoplasmic double-stranded RNA (dsRNA) sensor retinoic acid-inducible gene I (RIG-I) alone elicits a potent anti-tumor immune response resulting in melanoma control through the concurrent induction of a pro-inflammatory immune response and programmed cell death of tumor cells.[20–22] We recently reported that radioresistant p53-positive tumors can be controlled by intratumoral activation of RIG-I.[23] Here, we show that systemic BRAF/MEK inhibition synergizes with intratumoral RIG-I activation, inducing an immunogenic cell death in BRAF-mutated human and murine melanoma cells. *In vivo*, BRAF/MEK inhibitors act in concert with RIG-I ligands to synergistically improve the survival of melanoma-bearing mice. Our data suggest that intratumoral activation of RIG-I has the potential to improve cancer immunotherapy in almost half of patients with melanoma susceptible to BRAF/MEK inhibition.

RESULTS

Activation of RIG-I induces a pro-inflammatory signature and increased cell death of BRAF/MEK-inhibited melanoma

To investigate if the clinically relevant melanoma therapy of combined BRAF and MEK inhibition sensitizes cancer cells for the treatment with the RIG-I ligand 3pRNA, we treated human A375 (BRAF

Received 15 May 2024; accepted 16 July 2024;
https://doi.org/10.1016/j.omtn.2024.102283.

[4]These authors contributed equally

Correspondence: Rayk Behrendt, Institute for Clinical Chemistry and Clinical Pharmacology, University Hospital Bonn, Bonn, Germany.
E-mail: behrendt@uni-bonn.de
Correspondence: Gunther Hartmann, Institute for Clinical Chemistry and Clinical Pharmacology, University Hospital Bonn, Bonn, Germany.
E-mail: gunther.hartmann@uni-bonn.de

Published by Elsevier Inc. on behalf of The American Society of Gene and Cell Therapy.
This is an open access article under the CC BY license (http://creativecommons.org/licenses/by/4.0/).

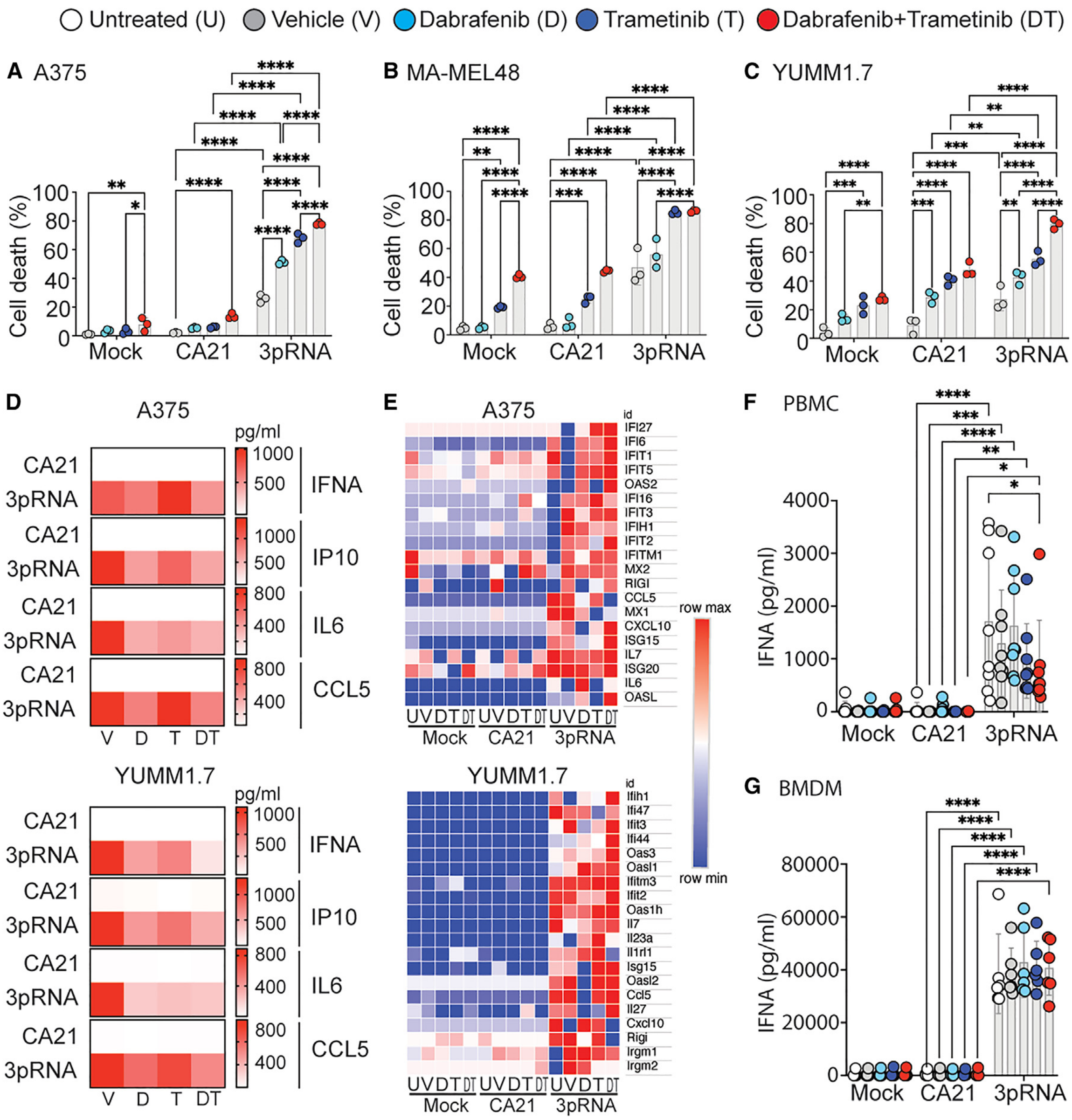

Figure 1. Combined BRAF/MEK inhibition synergizes with intratumoral RIG-I activation to kill melanoma cells and activate immune cells

In all images, treatment was as follows: untreated (U), DMSO (= vehicle [V]), 1,000 nM dabrafenib (D), 100 nM trametinib (T), and 1,000 nM dabrafenib +100 nM trametinib (DT). For RIG-I stimulation: untreated (MOCK), 1,000 ng/mL CA21 control RNA, 1,000 ng/mL 3pRNA. (A–C) Percentage of dead cells was quantified by flow cytometry after 48 h. Each data point represents one independent experiment shown as the mean of two technical replicates per experiment. Mean of the independent experiments ± SD is shown. (D) The indicated cytokines in the supernatant of A375 (top) and YUMM1.7 (bottom) cells after 24 h. Data represent the mean of five independent experiments with two technical replicates per experiment. (E) Transcription of interferon-regulated genes in A375 (top) and YUMM1.7 (bottom) cells was measured after 24 h by full-length mRNA sequencing. (F–G) IFNA protein in the supernatant of PBMCs (top) and bone marrow-derived macrophages (BMDMs) (bottom) was quantified by ELISA. Only significant results indicated. Each data point represents one biological donor. Per independent experiment, two technical replicates of two donors were analyzed. Mean of all donors ± SD is shown. Statistics for (A)–(C), (F), and (G) were calculated using two-way ANOVA with Tukey post hoc test. Only significant differences are labeled. *$p < 0.05$, **$p < 0.01$, ***$p < 0.001$, and ****$p < 0.0001$.

V600E mutated), Ma-Mel-48 (H-Ras G13I), and murine YUMM1.7 (BRAF V637E is a homolog to human V600E, V637E is sensitive to dabrafenib[24]) melanoma cell lines with dabrafenib and trametinib, alone or in combination. Dabrafenib alone did not increase the frequency of Annexin V- or 7AAD-positive A375 and Ma-Mel-48 cells, while trametinib alone had a moderate effect on the viability of Ma-Mel-48 cells (Figures 1A and 1B). Combined BRAF and MEK inhibition or transfection of 3pRNA alone only slightly increased the

frequency of dead A375 cells but killed almost half of Ma-Mel-48 melanoma cells (Figures 1A and 1B). The effect was synergistic when stimulation of RIG-I was paired with dabrafenib or trametinib alone, and it was strongest when all treatments were combined (Figures 1A and 1B). The findings were replicated in murine YUMM1.7 melanoma cells (Figure 1C).

Next, we screened the supernatant of BRAF/MEK-inhibited and control melanoma cells 24 h after 3pRNA stimulation for cytokine expression. We observed high levels of interferon alpha (IFNA), 10kDa interferon gamma-induced protein (IP10), interleukin-6 (IL6), and cc-chemokine ligand 5 (CCL5) proteins in the supernatant (Figure 1D) as well as a pro-inflammatory transcriptional signature (Figure 1E) in A375 and YUMM1.7 melanoma cells after transfection of 3pRNA, which was largely unaffected by dabrafenib or trametinib (Figures 1D and 1E). To investigate the effect of dabrafenib or trametinib on the activity of the RIG-I pathway in immune cells, we isolated human peripheral blood mononuclear cells (PBMCs) and murine bone marrow-derived macrophages and stimulated them with 3pRNA in the presence or absence of dabrafenib, trametinib, or their combination. Aside from a slightly diminished IFNA secretion in PBMCs in response to 3pRNA transfection, BRAF/MEK inhibition did not compromise the activity of RIG-I in professional primary immune cells (Figures 1F and 1G).

Combined BRAF/MEK inhibition overcomes resistance to 3pRNA treatment *in vivo*

To test the effect of RIG-I stimulation in combination with BRAF/MEK inhibition *in vivo*, we used a spontaneous melanoma model, in which tamoxifen-induced Cre-mediated expression of the BRAF V600E variant and concomitant deletion of the Pten tumor suppressor induces melanoma within 2–12 months after treatment.[25] We observed visible tumors in a substantial fraction of tamoxifen naive mice, which is in line with latest reports provided by the vendor (see Jax Strain 013590) but complicated harmonized treatment initiation of mice. Despite the leakiness, we induced tumor development in mice that had no visible tumor at the age of 6–8 weeks and started therapy 3 weeks later. Tumor progression was delayed by intratumoral 3pRNA injection in mice treated with dabrafenib and trametinib in combination, but the difference failed to reach statistical significance (Figure S1, $p = 0.0722$, unadjusted Gehan-Breslow-Wilcoxon test).

To overcome the uncontrollable biological variability introduced by Cre leakiness in the spontaneous tumor model, we turned toward a transplantation model using YUMM1.7 melanoma cells (Figure 2A). RIG-I agonist treatment started at a tumor diameter of maximal 3 mm twice a week for 21 days and daily treatment with dabrafenib and trametinib. At days 11–15 after treatment discontinuation, all surviving mice were re-challenged with YUMM1.7 cells injected into the contra-lateral side (Figure 2A). When analyzing the overall survival, irrespective of the re-challenge, YUMM1.7 tumors were resistant to trametinib and 3pRNA monotherapies but, as expected, sensitive to dabrafenib (Figure 2B). 3pRNA stimulation improved the survival of mice that received dabrafenib, but not trametinib monotherapy when compared to injections of CA21 control RNA. Strikingly, the clinically applied combination of dabrafenib and trametinib synergized most potently with additional stimulation of RIG-I, resulting in the strongest improvement in survival (Figure 2B). Censoring all events that are associated with the tumor re-challenge of mice that survived the therapy of the primary tumor confirmed the results of the overall analysis (Figure S1C). In contrast, analyzing only events that can be attributed to the contra-lateral tumor injection revealed no effect on survival in any treatment (Figure S1C). However, the latter analysis suffers from decreased power due to the low number of mice that survived the primary tumor.

Next, we analyzed the effect of the different therapeutic combinations on tumor-infiltrating immune cells and in tumor-draining lymph nodes 16 h after a single intratumoral injection of 3pRNA. Stimulation with the RIG-I agonist increased the frequencies of activated ($CD69^+$) $CD8^+$ cytotoxic T cells (CTL), natural killer (NK), and conventional dendritic cells (cDCs) in YUMM1.7 tumors (Figure 2C). Additional treatment with dabrafenib, trametinib, or both inhibitors combined did not suppress 3pRNA-induced local inflammation (Figure 2C). Similarly, higher frequencies of activated $CD8^+$ cells, NK cells, and cDCs were found in the tumor-draining lymph nodes, irrespective of the treatment with dabrafenib and trametinib (Figure 2D).

Collectively, we show that intratumoral injection of a synthetic RIG-I ligand led to the recruitment of activated immune cells into the tumor microenvironment and tumor-draining lymph nodes, which was unaltered by treatment with dabrafenib and trametinib. Most importantly, in this setting, intratumoral activation of RIG-I synergistically improved the survival of mice carrying BRAF-mutated melanoma.

DISCUSSION

Here, we show that intratumoral activation of intracellular nucleic acid sensor RIG-I[20,21] enhances the immune response and cell death of BRAF- and MEK-mutated melanoma cells. The effect was even stronger when combined with melanoma standard-of-care BRAF/MEK inhibitors dabrafenib and trametinib, respectively. The study supports previous observations, which suggested that inhibition of MAPKs sensitizes BRAF-mutated melanoma cells to RIG stimulation through the induction an IRF1-dependent pro-inflammatory program.[26] However, in clinical care of melanoma, BRAF and MEK inhibitors are strictly used in combination, while dabrafenib monotherapy is a rare exception. We now also show that the clinically applied combination of BRAF/MEK inhibition does not compromise the therapeutic effects of intratumoral RIG-I activation in *in vivo* melanoma models: first, 3pRNA directly acts within the tumor cells, likely inducing a caspase-dependent cell death as observed previously,[23] thereby reducing tumor burden. Second, activation of RIG-I induced a pro-inflammatory response in the tumor cells, thereby attracting immune cells to the site of the tumor. Third, RIG-I agonists directly activate professional antigen-presenting cells of the host that reside in the tumor, which subsequently instruct adaptive anti-tumor immunity. We observed that both the tumor cell-intrinsic and immune cell-intrinsic RIG-I responses are functional under combined BRAF/MEK inhibitor therapy.

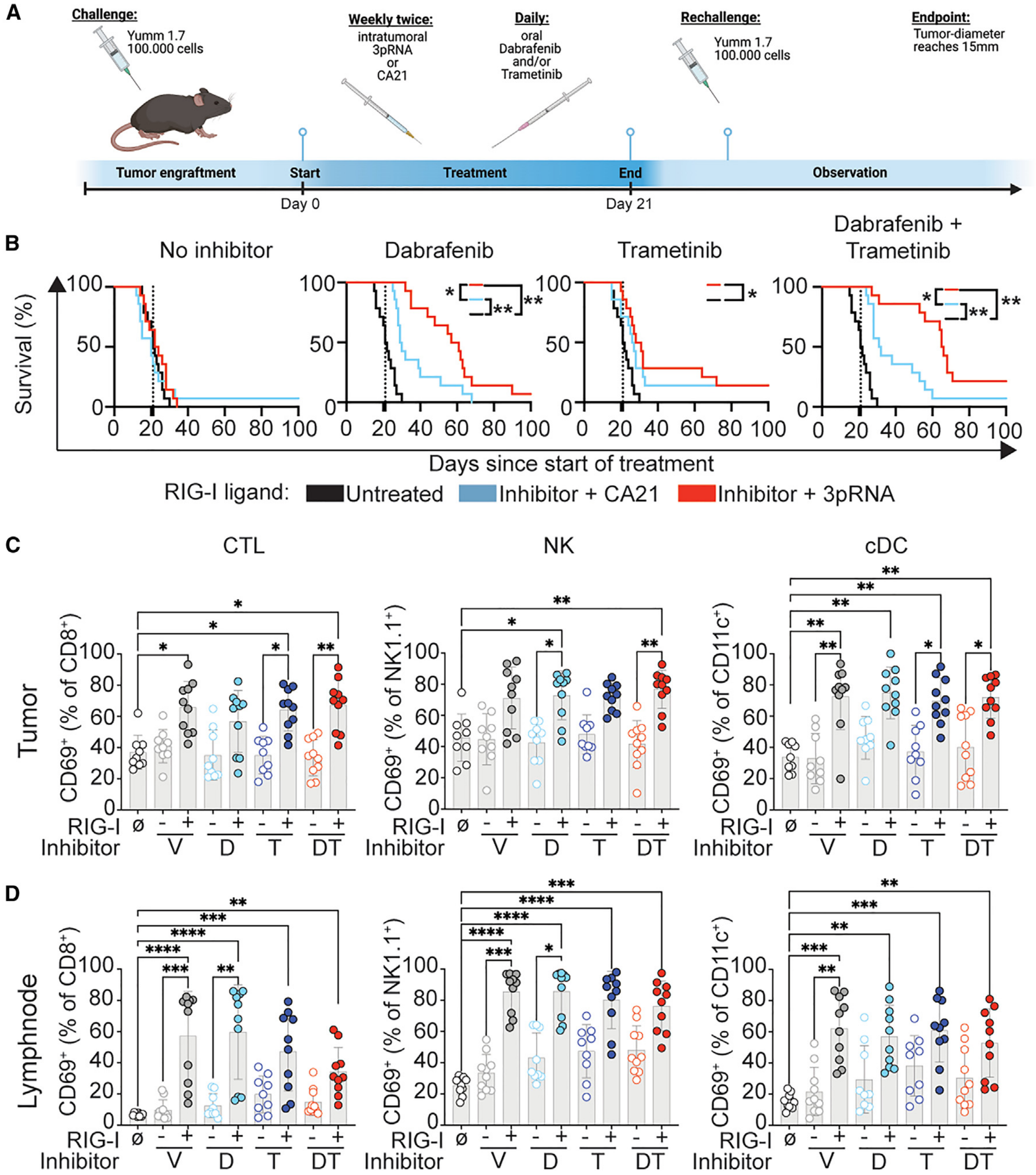

Figure 2. Synergistic anti-tumor immunity against BRAF-mutated melanoma *in vivo* through combined RIG-I stimulation and BRAF/MEK inhibition

(A) Timeline of YUMM1.7 transplantation, treatment, and analysis. (B) Overall survival of tumor-bearing mice treated as indicated. Three independent experiments were performed with a total $n = 14$ in each group. Log-rank Mantel-Cox test with Holm-Šídák correction for 12 multiple comparisons. $^{*}p < 0.05$ and $^{**}p < 0.01$. (C and D) Frequency of activated ($CD69^{+}$) among $CD45^{+}CD4^{-}CD8^{+}$ CTLs, $CD45^{+}NK1.1^{+}$ NK cells, and $CD45^{+}CD11b^{+}CD11c^{+}MHC$ class II^{+} cDCs in the tumor (C) and the tumor-draining lymph node (D) 16 h after a single treatment. Mean ± SD is shown. Kruskal-Wallis test with Dunn's correction for 16 multiple comparisons. $^{*}p < 0.05$, $^{**}p < 0.01$, $^{***}p < 0.001$, and $^{****}p < 0.0001$. Only significant results indicated.

In vivo, intratumoral RIG-I stimulation could overcome the resistance of YUMM1.7 to trametinib monotherapy, and it significantly prolonged survival under dabrafenib therapy alone and when combined with trametinib. Of note, YUMM1.7 melanoma are by far less immunogenic when compared to RIG-I-sensitive YUMMER 1.7 melanoma,[27] and we provide evidence that combined BRAF/MEK inhibition renders even YUMM1.7 melanoma susceptible to RIG-I agonist-based immunotherapy. We previously observed that

RIG-I stimulation overcomes radiotherapy resistance in p53-positive melanoma, suggesting that RIG-I-driven innate immune activation in the tumor microenvironment might represent a general strategy to enhance various melanoma therapies.[23]

Taken together, we show that intratumoral stimulation of RIG-I with its synthetic ligand 3pRNA in combination with the clinically relevant BRAF/MEK double inhibition outperforms the therapeutic effect of the individual therapies against BRAF-mutated melanoma *in vivo*. Our data suggest that this combination, which potentially improves the outcome for almost half of patients with melanoma, could be a major asset to the toolbox of melanoma therapy.

MATERIAL AND METHODS

Standard procedures are described in the supplemental information.

Mouse experiments

All mouse experiments were approved by the Landesamt für Natur, Umwelt und Verbraucherschutz NRW (81-02.04.2020.A156). B6.Cg-Tg(Tyr-cre/ERT2)13Bos $Braf^{tm1Mmcm}$ $Pten^{tm1Hwu}$/BosJ ($BRaf^{CA}$, $Pten^{loxP}$, Tyr:$CreER^{T2}$) were purchased from Jackson Laboratory (JAX 013590).[25] Mice of both sexes were topically treated with 2 μL of 5 mM 4-hydroxytamoxifen (Sigma-Aldrich) at the age of 6–8 weeks and observed as reported in the main text. For transplantation of YUMM1.7 cells, 100,000 cells in 50 μL of Dulbecco's balanced salt solution (PBS, Thermo Fisher Scientific) were intracutaneously injected in the flank of C56BL6/J mice. When tumor diameters reached 2–3 mm, mice were randomly assigned to treatment groups, and tumor diameter was measured daily using a caliper.

BRAF inhibitor dabrafenib (mesylate) and MEK inhibitor trametinib (both MedChem Express) were suspended in an aqueous solution containing 0.5% (w/v) hydroxypropyl-methylcellulose (Sigma-Aldrich) and 0.2% (v/v) Tween 80 (Sigma-Aldrich). Inhibitors were administered by oral gavage in a final volume of 100 μL. To stimulate RIG-I 3pRNA or CA21, control RNA was complexed with jetPEI (Polyplus Transfection) according to the manufacturer's protocol and injected intratumorally.

Treatment of cells

For RIG-I stimulation, a chemically synthesized dsRNA with a 3p end, as previously described, was used.[28] CA21 control RNA was synthesized at Biomers (5′-CACACACACACACACACACAC-3′). For *in vitro* transfection, RNA was complexed to Lipofectamine 2000 (Invitrogen) according to the manufacturer's protocol (Thermo Fisher Scientific).[23]

For *in vitro* experiments, dabrafenib (MedChem Express) and trametinib (Selleck Chemicals) were dissolved in dimethyl sulfoxide (DMSO) (Carl Roth) and added at indicated concentrations. DMSO at the highest used concentration served as the vehicle control.

Statistical analysis

Statistical analysis was calculated with Prism 9 (v.9.4.1) software (GraphPad Software). The respective tests are indicated in the figure legends, with $p < 0.05$ considered to be statistically significant.

DATA AND CODE AVAILABILITY

All primary data are available upon request. Gene expression data have been deposited at the GEO database.

SUPPLEMENTAL INFORMATION

Supplemental information can be found online at https://doi.org/10.1016/j.omtn.2024.102283.

ACKNOWLEDGMENTS

This study was funded by Deutsche Forschungsgemeinschaft (DFG, German Research Foundation) – Project-ID 369799452 – TRR237 Nucleic Acid Immunity, project A04 to G.H. and B19 to R.B. and EXC 2151: ImmunoSensation2 to G.H., J.L.S.B., and R.B. C.G. was supported by the BonnNi MD program for medical students funded by the Else Kroner-Fresenius Foundation. The graphical abstract was created with BioRender.

AUTHOR CONTRIBUTIONS

C.G., Y.P.T., P.M., M.J., and M.R. performed experiments. C.G., Y.P.T., M.J., T.M.S.-G., J.L.S.-B., and R.B. analyzed data. M.R., R.B., and G.H. conceptualized and supervised the study. C.G., R.B., and G.H. wrote the initial version of the manuscript.

DECLARATION OF INTERESTS

G.H. is an inventor on a patent covering synthetic RIG-I ligands. M.R. and G.H. were co-founders of Rigontec GmbH. J.L.S.-B. is a co-founder and shareholder of LAMPseq Diagnostics.

REFERENCES

1. Siegel, R.L., Giaquinto, A.N., and Jemal, A. (2024). Cancer statistics, 2024. CA A Cancer J. Clin. *74*, 12–49. https://doi.org/10.3322/caac.21820.
2. Whiteman, D.C., Green, A.C., and Olsen, C.M. (2016). The Growing Burden of Invasive Melanoma: Projections of Incidence Rates and Numbers of New Cases in Six Susceptible Populations through 2031. J. Invest. Dermatol. *136*, 1161–1171. https://doi.org/10.1016/j.jid.2016.01.035.
3. Schadendorf, D., van Akkooi, A.C.J., Berking, C., Griewank, K.G., Gutzmer, R., Hauschild, A., Stang, A., Roesch, A., and Ugurel, S. (2018). Melanoma. Lancet Lond. Engl. *392*, 971–984. https://doi.org/10.1016/S0140-6736(18)31559-9.
4. Roberts, P.J., and Der, C.J. (2007). Targeting the Raf-MEK-ERK mitogen-activated protein kinase cascade for the treatment of cancer. Oncogene *26*, 3291–3310. https://doi.org/10.1038/sj.onc.1210422.
5. Samatar, A.A., and Poulikakos, P.I. (2014). Targeting RAS-ERK signalling in cancer: promises and challenges. Nat. Rev. Drug Discov. *13*, 928–942. https://doi.org/10.1038/nrd4281.
6. Cancer Genome Atlas Network (2015). Genomic Classification of Cutaneous Melanoma. Cell *161*, 1681–1696. https://doi.org/10.1016/j.cell.2015.05.044.
7. Long, G.V., Menzies, A.M., Nagrial, A.M., Haydu, L.E., Hamilton, A.L., Mann, G.J., Hughes, T.M., Thompson, J.F., Scolyer, R.A., and Kefford, R.F. (2011). Prognostic and clinicopathologic associations of oncogenic BRAF in metastatic melanoma. J. Clin. Oncol. *29*, 1239–1246. https://doi.org/10.1200/JCO.2010.32.4327.
8. Long, G.V., Hauschild, A., Santinami, M., Atkinson, V., Mandalà, M., Chiarion-Sileni, V., Larkin, J., Nyakas, M., Dutriaux, C., Haydon, A., et al. (2017). Adjuvant

Dabrafenib plus Trametinib in Stage III BRAF-Mutated Melanoma. N. Engl. J. Med. *377*, 1813–1823. https://doi.org/10.1056/NEJMoa1708539.

9. Hauschild, A., Dummer, R., Schadendorf, D., Santinami, M., Atkinson, V., Mandalà, M., Chiarion-Sileni, V., Larkin, J., Nyakas, M., Dutriaux, C., et al. (2018). Longer Follow-Up Confirms Relapse-Free Survival Benefit With Adjuvant Dabrafenib Plus Trametinib in Patients With Resected BRAF V600-Mutant Stage III Melanoma. J. Clin. Oncol. *36*, 3441–3449. https://doi.org/10.1200/JCO.18.01219.
10. Long, G.V., Stroyakovskiy, D., Gogas, H., Levchenko, E., de Braud, F., Larkin, J., Garbe, C., Jouary, T., Hauschild, A., Grob, J.J., et al. (2014). Combined BRAF and MEK inhibition versus BRAF inhibition alone in melanoma. N. Engl. J. Med. *371*, 1877–1888. https://doi.org/10.1056/NEJMoa1406037.
11. Robert, C., Karaszewska, B., Schachter, J., Rutkowski, P., Mackiewicz, A., Stroiakovski, D., Lichinitser, M., Dummer, R., Grange, F., Mortier, L., et al. (2015). Improved overall survival in melanoma with combined dabrafenib and trametinib. N. Engl. J. Med. *372*, 30–39. https://doi.org/10.1056/NEJMoa1412690.
12. Avery, T.Y., Köhler, N., Zeiser, R., Brummer, T., and Ruess, D.A. (2022). Onco-immunomodulatory properties of pharmacological interference with RAS-RAF-MEK-ERK pathway hyperactivation. Front. Oncol. *12*, 931774. https://doi.org/10.3389/fonc.2022.931774.
13. Hu-Lieskovan, S., Robert, L., Homet Moreno, B., and Ribas, A. (2014). Combining targeted therapy with immunotherapy in BRAF-mutant melanoma: promise and challenges. J. Clin. Oncol. *32*, 2248–2254. https://doi.org/10.1200/JCO.2013.52.1377.
14. Kuske, M., Westphal, D., Wehner, R., Schmitz, M., Beissert, S., Praetorius, C., and Meier, F. (2018). Immunomodulatory effects of BRAF and MEK inhibitors: Implications for Melanoma therapy. Pharmacol. Res. *136*, 151–159. https://doi.org/10.1016/j.phrs.2018.08.019.
15. Garbe, C., Amaral, T., Peris, K., Hauschild, A., Arenberger, P., Basset-Seguin, N., Bastholt, L., Bataille, V., del Marmol, V., Dréno, B., et al. (2022). European consensus-based interdisciplinary guideline for melanoma. Part 2: Treatment - Update 2022. Eur. J. Cancer *170*, 256–284. https://doi.org/10.1016/j.ejca.2022.04.018.
16. Dixon-Douglas, J.R., Patel, R.P., Somasundram, P.M., and McArthur, G.A. (2022). Triplet Therapy in Melanoma - Combined BRAF/MEK Inhibitors and Anti-PD-(L)1 Antibodies. Curr. Oncol. Rep. *24*, 1071–1079. https://doi.org/10.1007/s11912-022-01243-x.
17. Haist, M., Stege, H., Kuske, M., Bauer, J., Klumpp, A., Grabbe, S., and Bros, M. (2023). Combination of immune-checkpoint inhibitors and targeted therapies for melanoma therapy: The more, the better? Cancer Metastasis Rev. *42*, 481–505. https://doi.org/10.1007/s10555-023-10097-z.
18. Ribas, A., Hodi, F.S., Callahan, M., Konto, C., and Wolchok, J. (2013). Hepatotoxicity with combination of vemurafenib and ipilimumab. N. Engl. J. Med. *368*, 1365–1366. https://doi.org/10.1056/NEJMc1302338.
19. Minor, D.R., Puzanov, I., Callahan, M.K., Hug, B.A., and Hoos, A. (2015). Severe gastrointestinal toxicity with administration of trametinib in combination with dabrafenib and ipilimumab. Pigment Cell Melanoma Res. *28*, 611–612. https://doi.org/10.1111/pcmr.12383.
20. Besch, R., Poeck, H., Hohenauer, T., Senft, D., Häcker, G., Berking, C., Hornung, V., Endres, S., Ruzicka, T., Rothenfusser, S., and Hartmann, G. (2009). Proapoptotic signaling induced by RIG-I and MDA-5 results in type I interferon-independent apoptosis in human melanoma cells. J. Clin. Invest. *119*, 2399–2411. https://doi.org/10.1172/JCI37155.
21. Poeck, H., Besch, R., Maihoefer, C., Renn, M., Tormo, D., Morskaya, S.S., Kirschnek, S., Gaffal, E., Landsberg, J., Hellmuth, J., et al. (2008). 5'-Triphosphate-siRNA: turning gene silencing and Rig-I activation against melanoma. Nat. Med. *14*, 1256–1263. https://doi.org/10.1038/nm.1887.
22. Duewell, P., Steger, A., Lohr, H., Bourhis, H., Hoelz, H., Kirchleitner, S.V., Stieg, M.R., Grassmann, S., Kobold, S., Siveke, J.T., et al. (2014). RIG-I-like helicases induce immunogenic cell death of pancreatic cancer cells and sensitize tumors toward killing by CD8(+) T cells. Cell Death Differ. *21*, 1825–1837. https://doi.org/10.1038/cdd.2014.96.
23. Lambing, S., Tan, Y.P., Vasileiadou, P., Holdenrieder, S., Müller, P., Hagen, C., Garbe, S., Behrendt, R., Schlee, M., van den Boorn, J.G., et al. (2023). RIG-I immunotherapy overcomes radioresistance in p53-positive malignant melanoma. J. Mol. Cell Biol. *15*, mjad001. https://doi.org/10.1093/jmcb/mjad001.
24. Lasolle, H., Schiavo, A., Tourneur, A., Gillotay, P., de Faria da Fonseca, B., Ceolin, L., Monestier, O., Aganahi, B., Chomette, L., Kizys, M.M.L., et al. (2024). Dual targeting of MAPK and PI3K pathways unlocks redifferentiation of Braf-mutated thyroid cancer organoids. Oncogene *43*, 155–170. https://doi.org/10.1038/s41388-023-02889-y.
25. Dankort, D., Curley, D.P., Cartlidge, R.A., Nelson, B., Karnezis, A.N., Damsky, W.E., You, M.J., DePinho, R.A., McMahon, M., and Bosenberg, M. (2009). Braf(V600E) cooperates with Pten loss to induce metastatic melanoma. Nat. Genet. *41*, 544–552. https://doi.org/10.1038/ng.356.
26. Brägelmann, J., Lorenz, C., Borchmann, S., Nishii, K., Wegner, J., Meder, L., Ostendorp, J., Ast, D.F., Heimsoeth, A., Nakasuka, T., et al. (2021). MAPK-pathway inhibition mediates inflammatory reprogramming and sensitizes tumors to targeted activation of innate immunity sensor RIG-I. Nat. Commun. *12*, 5505. https://doi.org/10.1038/s41467-021-25728-8.
27. Jiang, X., Muthusamy, V., Fedorova, O., Kong, Y., Kim, D.J., Bosenberg, M., Pyle, A.M., and Iwasaki, A. (2019). Intratumoral delivery of RIG-I agonist SLR14 induces robust antitumor responses. J. Exp. Med. *216*, 2854–2868. https://doi.org/10.1084/jem.20190801.
28. Marx, S., Kümmerer, B.M., Grützner, C., Kato, H., Schlee, M., Renn, M., Bartok, E., and Hartmann, G. (2022). RIG-I-induced innate antiviral immunity protects mice from lethal SARS-CoV-2 infection. Mol. Ther. Nucleic Acids *27*, 1225–1234. https://doi.org/10.1016/j.omtn.2022.02.008.

OMTN, Volume *35*

Supplemental information

Synthetic RIG-I agonist-mediated cancer immunotherapy synergizes with MAP kinase inhibition against BRAF-mutated melanoma

Christian Grützner, Yu Pan Tan, Patrick Müller, Thais M. Schlee-Guimaraes, Marius Jentzsch, Jonathan L. Schmid-Burgk, Marcel Renn, Rayk Behrendt, and Gunther Hartmann

Supplemental Figure

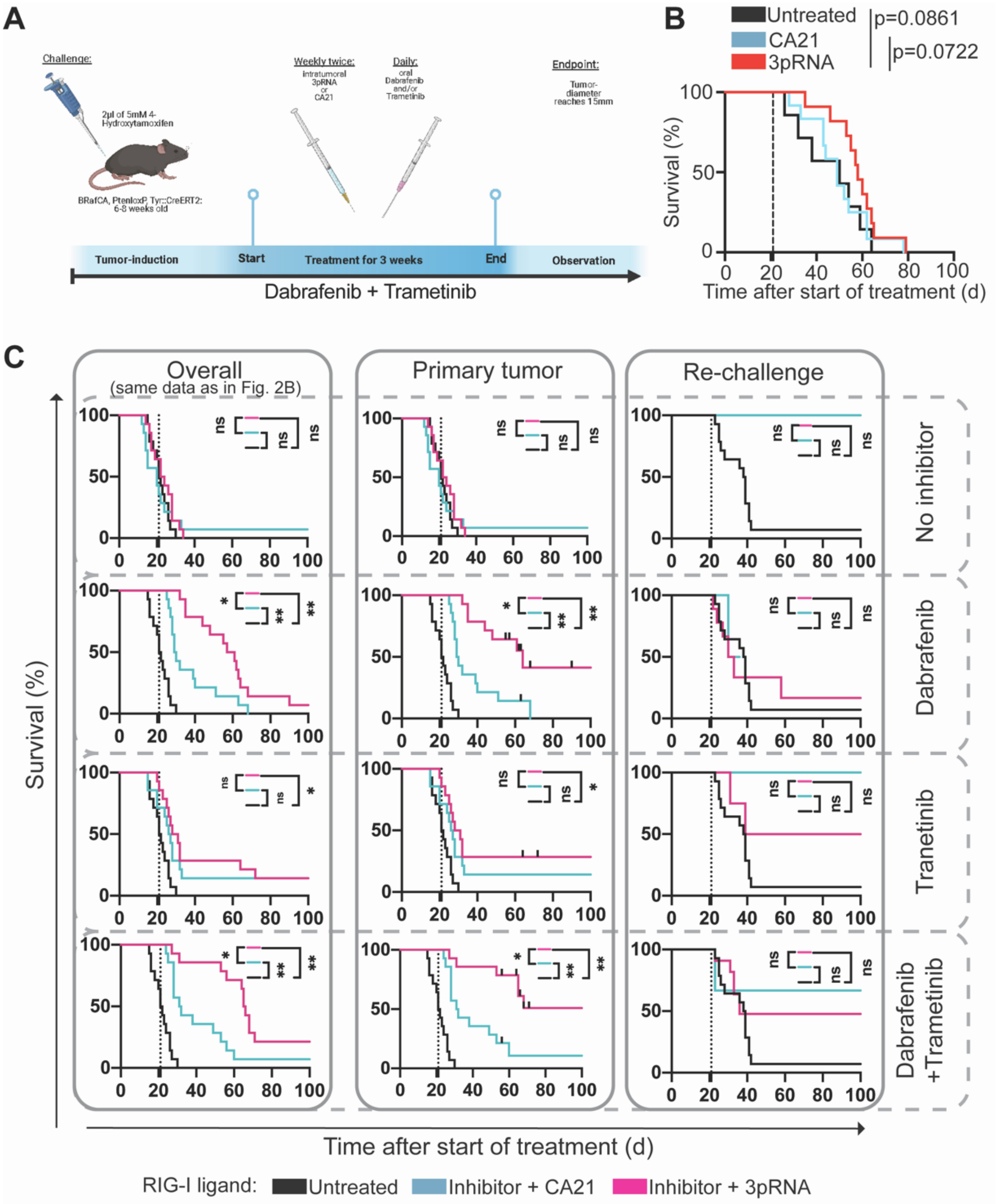

Figure S1: In vivo effect of 3pRNA in combination with Dabrafenib and Trametinib on the survival of melanoma-bearing mice. A. Overview of the experimental design for induction and treatment of Tamoxifen-induced Braf/Pten-mutated melanoma. B. Kaplan-Meyer analysis of Tamoxifen-treated mice that received Dabrafenib and Trametinib and 3pRNA (n=11), CA21-control RNA (n=12) or were left untreated (n=7). Treatment was stopped at day 21 (dotted line). Statistics unadjusted Gehan-Breslow-Wilcoxon test. C) Survival analysis of mice that were inoculated with YUMM1.7 melanoma cells and treated according to the scheme shown in Fig. 2A. Three independent experiments were performed with a total n=14 in each group. Survival curves were generated looking at the overall survival ("Overall", display identical to Fig. 2B), taking only events that could be attributed to the primary tumor into account ("Primary tumor") or only considering events that were presumably caused by the re-challenge tumor ("Re-challenge"). Log-rank Mantel-Cox test with Holm-Šídák correction for 12 multiple comparisons. * - p<0.05; ** - p<0.01.

Supplemental Methods

Cell lines

Human A375 melanoma cell line was kindly provided by Michael Hölzel (University Hospital Bonn, Germany) and Ma-Mel-48 melanoma were kindly provided by the Department of Dermatology at University Hospital Bonn. Murine Yale University Mouse Melanoma - YUMM1.7 was purchased from ATCC [1]. All cell lines were incubated at 37°C 5% CO2 and regularly checked for mycoplasma contamination. A375 cell line was cultured in DMEM with 10 % (v/v) fetal calf serum (FCS) and 100 units penicillin and 100 µg streptomycin per mL Media (p/s) (all Thermo Fisher Scientific). Ma-Mel-48 cell were cultured in RPMI (Thermo Fisher Scientific) containing the same supplements as A375. YUMM cell lines were cultured in DMEM/F-12, GlutaMAX™ (Thermo Fisher Scientific) supplemented with FCS, p/s and non-essential amino acids (Thermo Fisher Scientific).

Peripheral blood mononuclear cell experiments (PBMC):

All experiments on human samples were approved by local ethical committee (Ethikkommission der Medizinischen Fakultät Bonn - File Reference: 515/20). PBMC were isolated by Ficoll (GE Healthcare/Cytiva). Freshly isolated cells were plated in 96 well flat bottom cell culture plates at concentration of 200.000 per well in RPMI 1640 Medium with 10% FCS and 100 units penicillin and 100 µg streptomycin per mL. Cells were stimulated as described in the main text.

Murine bone marrow derived macrophages (BMDM):

Murine bone marrow derived macrophages (BMDM) were differentiated from C56BL/6N whole bone marrow cells for seven days in RPMI + 30% L929 supernatant. 100.000 cells were seeded in 96-well flat bottom tissue culture plates in RPMI as described above RPMI + 15% L929 supernatant and stimulated as described above. All experiments on mouse organs were approved by lokal ethical committee.

Apoptosis assessment by flowcytometry

20.000 (A375/ Ma-Mel-48) or 10.000 (YUMM1.7) cells per well were plated in 96-well flat bottom tissue culture plates. The next day cells were stimulated and/or co-incubated with inhibitors at indicated concentrations. For assessment of apoptosis cells were trypsinized (0.25%, Thermo Fischer Scientific) and stained with Annexin V Alexa Fluor® 647 (BioLegend) at dilution of 1:30 in Annexin binding buffer (10 mM HEPES, 140 mM NaCl, 2.5 mM $CaCl_2$) for 15 min in the dark. Cells were washed in FACS buffer and kept resuspended annexin binding buffer. Shortly before measurement via Attune NxT flow cytometer (Thermo Fisher Scientific) 7-Aminoactinomycin D (Enzo Life Sciences) at final concentration of 1,25 µg/mL was added.

Enzyme linked immuno assay (ELISA):

Commercial ELISAs (Human IP-10, BD; human IFNA, Thermo Fisher) were performed using half of the amounts of chemicals recommended by the manufacturers.

A self-made murine IFNA ELISA was performed in half-area 96 well-microplate (clear, microlon, high binding from Greiner Bio-One) utilizing anti-Mouse IFN-alpha (clone RMMA-1 (MAb)), mouse IFN-alpha A and anti-Mouse IFN-alpha (rabbit Serum (PAb)) (all PBL Assay Science).

Muliplex cytokine Assays:

LEGENDplex™ Assays (BioLegend) were used according to manufacturer's recommendations but run in a 384-well assay plate with volumes adjusted accordingly as previously described [2]. Human cytokines were quantified with the COVID-19 Cytokine Storm Panel (14-plex) (BioLegend 741089) and murine cytokines using Mouse Anti-Virus Response Panel (13-plex) (BioLegend 740622) were used. Data were analyzed using cloud-based LEGENDplex™ Data Analysis Software Suite.

Flow cytometry:

Tumors were desintegrated in PBS containing 1mg/mL Collagenase D (Roche) and 5 % FCS for 20 minutes at 37°C and afterwards passed through a 70 µm cell strainer. Tumor draining lymph nodes were passed through a cell strainer. Zombie Aqua™ Fixable Viability Kit (1:500 in PBS, BioLegend) was added for 20 min at room temperature followed by Fc block using CD16/32 antibodies (1:200, eBioscience) for another 20 mins. Surface marker staining was performed for 20 minutes at room temperature in the dark with following antibodies all diluted at 1:200 in FACS-buffer (PBS with 10% FCS, 2 mM EDTA and 0.05% sodium azide). Results were calculated based on the following gatings using FlowJo software version 10.8.1(BD). 1. pre-gating: SSC:FSC > FSC-H:FSC-A > CD45-PerCP^{+}:Zombie-negative. For identification of immune cell subsets subsequent gatings were applied for activated CD8-T-cells: CD69-AF647^{+} : CD8a-BV785^{+}; activated NK-cells: CD69-AF647^{+} : NK1.1-BV650^{+}., and for cDCs: CD11b-BV650^{+} : FSC > CD11c-BV421^{dim} : MHCII-AF488^{+}. In these subsets also the CD86-BV785 signal was quantified.

RNA-Seq-analysis:

RNA was extracted with RNeasy Mini Kit (Qiagen) used according to the manufacturers protocol. mRNA was purified by poly-A enrichment using NEBNext® Poly(A) mRNA Magnetic Isolation Module. For library preparation NEBNext® Ultra™ II Directional RNA Library Prep with Sample Purification Beads with NEBNext® Multiplex Oligos for Illumina® were used. Sequencing was performed on an Illumina NextSeq 2000 using a P2 100-cycle kit. Sequencing reads were aligned to the human (GRCh38) and Mus musculus (GRCm39) reference genome using STAR (Dobin et al., 2013). and quantified with HTSeq2.0 (G Putri, S Anders, PT Pyl, JE Pimanda, F Zanini Analysing high-throughput sequencing data in Python with HTSeq 2.0 [3]. Expression analysis was performed with the statical R-package edgeR [4] Library sizes across samples were normalized using TMM (trimmed mean of M values).
Data have been deposited in the GEO database under the accession numbers GSE269008 and GSE268982 for mouse and human cell lines, respectively. Heatmaps display log-counts-per-million values of the indicated transcripts after normalization. Heatmaps were generated using the mighty Morpheus (https://software.broadinstitute.org/morpheus).

References for supplemental methods

1. Meeth, K., Wang, J.X., Micevic, G., Damsky, W., and Bosenberg, M.W. (2016). The YUMM lines: a series of congenic mouse melanoma cell lines with defined genetic alterations. Pigment Cell Melanoma Res. *29*, 590–597. https://doi.org/10.1111/pcmr.12498.

2. Lambing, S., Holdenrieder, S., Müller, P., Tan, Y.P., Hagen, C., Garbe, S., Schlee, M., Boorn, J.G. van den, Bartok, E., Hartmann, G., et al. (2022). RIG-I immunotherapy overcomes radioresistance in p53-positive malignant melanoma. Preprint at bioRxiv, https://doi.org/10.1101/2021.10.16.464638 https://doi.org/10.1101/2021.10.16.464638.

3. Putri, G.H., Anders, S., Pyl, P.T., Pimanda, J.E., and Zanini, F. (2022). Analysing high-throughput sequencing data in Python with HTSeq 2.0. Bioinformatics *38*, 2943–2945. https://doi.org/10.1093/bioinformatics/btac166.

4. Robinson, M.D., McCarthy, D.J., and Smyth, G.K. (2010). edgeR: a Bioconductor package for differential expression analysis of digital gene expression data. Bioinforma. Oxf. Engl. *26*, 139–140. https://doi.org/10.1093/bioinformatics/btp616.
